# Serum Neuron-Specific Enolase as a Predictor of Neurological Outcomes at Hospital Discharge in Post-Cardiac Arrest Patients: A Prospective Study

**DOI:** 10.7759/cureus.99748

**Published:** 2025-12-21

**Authors:** Raluca Badila, Mihai Sava, Alina S Bereanu, Sandra Neamtu, Simina Mustatea, Corina Roman-Filip

**Affiliations:** 1 Anesthesia and Critical Care, County Clinical Emergency Hospital of Sibiu, Sibiu, ROU; 2 Pulmonology, Clinical Pneumology Hospital of Sibiu, Sibiu, ROU; 3 Neurology, County Clinical Emergency Hospital of Sibiu, Sibiu, ROU

**Keywords:** biomarkers, cardiac arrest, neurological outcome, neuron specific enolase, prognostication

## Abstract

Background

Post-cardiac arrest encephalopathy is a major determinant of outcome in patients who achieve return of spontaneous circulation (ROSC). Despite advances in post-resuscitation care, accurately predicting neurological prognosis remains challenging. Serum neuron-specific enolase (NSE) is a validated biomarker of hypoxic-ischemic brain injury, although optimal time points and thresholds require further clinical evaluation.

Objective

This prospective study assessed the prognostic value of serum NSE measured at 24 and 72 hours after cardiac arrest in predicting neurological outcome at hospital discharge using the Cerebral Performance Category (CPC) scale.

Methods

We conducted a prospective observational study at County Emergency Clinical Hospital of Sibiu over a 12-month period (February 2024-February 2025). A total of 135 patients with post-cardiac arrest syndrome were admitted, of whom 120 met the inclusion criteria. Serum NSE levels were measured at 24 and 72 hours post-ROSC. Neurological outcome at discharge was categorized as favorable (Cerebral Performance Category, (CPC 1-2)) or unfavorable (CPC 3-5). Discrimination was evaluated using receiver operating characteristic (ROC) curves, and optimal cutoff values were identified using Youden’s index.

Results

Of the 120 included patients, 20 (17%) survived to hospital discharge. Median NSE concentrations at both 24 and 72 hours were significantly higher in patients with unfavorable outcomes (p< 0.001). NSE demonstrated good discriminative accuracy, with an AUC of 0.80 at 24 hours and 0.89 at 72 hours. The optimal prognostic cutoff at 72 hours was 68 ng/mL, yielding 82% sensitivity and 90% specificity for predicting an unfavourable outcome.

Conclusions

Serum NSE levels measured 24-72 hours after cardiac arrest are a reliable predictor of neurological outcome at hospital discharge. The strongest discriminative performance was observed at 72 hours. Incorporating NSE into a multimodal prognostication framework may enhance early risk assessment and guide clinical decision-making in post-cardiac arrest care.

## Introduction

Post-cardiac arrest syndrome remains a major cause of morbidity and mortality among patients who achieve return of spontaneous circulation (ROSC) after cardiac arrest. Despite advancements in resuscitation science, targeted post-resuscitation care, and standardized prognostication algorithms, neurological injury continues to be the determinant factor influencing survival and long-term functional outcome. Early and accurate prediction of neurological prognosis is essential for guiding clinical decision-making, resource allocation, and discussions with families regarding expected outcomes [1-3].

Hypoxic-ischemic brain injury following cardiac arrest triggers a cascade of metabolic, inflammatory, and apoptotic mechanisms that contribute to delayed neuronal damage. Conventional clinical examination is often unreliable in the early post-arrest period due to sedation, metabolic disturbances, and therapeutic interventions [4]. Consequently, international guidelines emphasize a multimodal approach, incorporating neurological examination, electrophysiology, neuroimaging, and biochemical markers [5].

Among available biomarkers, neuron-specific enolase (NSE) has emerged as one of the most extensively studied serum indicators of neuronal injury. Elevated concentrations of NSE correlate with the severity of hypoxic-ischemic damage and have shown prognostic value for predicting neurological outcomes in both observational cohorts and controlled trials [6,7]. Nevertheless, several limitations persist, including variability related to hemolysis, renal clearance, timing of sampling, and inter-assay differences. Furthermore, although multiple studies highlight the discriminative performance of NSE at 48-72 hours, optimal thresholds and accuracy vary across populations and clinical settings [8-10]. Also, numerous studies highlighted the prognostic value of biomarkers in cerebrovascular disease. These findings are highly relevant to post-cardiac arrest hypoxic-ischemic brain injury, as both conditions share common mechanisms such as ischemia-reperfusion injury and inflammation-driven secondary neuronal damage [11].

Given these uncertainties, further prospective data are needed to clarify the prognostic performance of NSE in real-world clinical practice. The present study aims to evaluate the predictive value of serum NSE measured at 24 and 72 hours after ROSC in determining neurological outcome at hospital discharge among adult patients admitted after cardiac arrest to County Emergency Clinical Hospital of Sibiu.

## Materials and methods

Study design 

This prospective observational study was conducted at County Emergency Clinical Hospital of Sibiu, a tertiary care centre serving both urban and rural populations in central Romania. The study period extended over 12 months, from February 2024 to February 2025. All consecutive adult patients admitted after return of spontaneous circulation (ROSC) following cardiac arrest were screened for eligibility.

Study population

A total of 135 patients with post-cardiac arrest syndrome were admitted during the study period. Of these, 120 patients met the inclusion criteria and were enrolled in the final analysis.

Inclusion and exclusion criteria

Eligible patients were adults aged 18 years or older who achieved sustained return of spontaneous circulation (ROSC) following either out-of-hospital or in-hospital cardiac arrest. Only individuals who remained comatose after ROSC, defined by a Glasgow Coma Scale score of 8 or less, were considered for enrollment. To ensure consistency in biomarker assessment, inclusion required the availability of serum neuron-specific enolase (NSE) measurements at both 24 and 72 hours after ROSC, obtained using an institutional standardized electrochemiluminescence immunoassay protocol (Roche Diagnostics, Cobas e411), according to the manufacturer’s instructions.

Patients were excluded from analysis if they died within the first 24 hours after ROSC, before the initial NSE sample could be collected. Additional exclusion criteria included laboratory-confirmed severe hemolysis that could interfere with the accuracy of NSE quantification, the absence of NSE values at either of the two predefined time points, and the presence of significant traumatic brain injury that could act as an alternative cause for neurological impairment. Individuals with known malignancies associated with elevated NSE levels, such as small-cell lung cancer, were also excluded to avoid confounding effects on biomarker interpretation.

Data collection and variables

Clinical and demographic data were prospectively recorded for all enrolled patients. Variables included age, sex, and documented comorbidities, particularly pre-existing cardiovascular disease. Information regarding diabetes mellitus and chronic kidney disease was inconsistently available and was therefore not systematically included in the baseline analysis. Cardiac arrest-related characteristics were comprehensively documented, with particular attention to the type of arrest (out-of-hospital or in-hospital), the initial cardiac rhythm at presentation, and the quality of resuscitation efforts. Information regarding post-resuscitation interventions, including targeted temperature management protocols, hemodynamic support, and ventilation strategies, was obtained from electronic medical records.

Serum NSE levels were measured at two standardized intervals-24 hours and 72 hours after ROSC-using a validated electrochemiluminescence immunoassay performed in the hospital’s central laboratory. All samples were processed according to strict institutional procedures to minimize preanalytical variability, including prompt centrifugation, avoidance of hemolysis, and controlled storage conditions until analysis. These measurements provided the basis for examining temporal changes in NSE levels and their association with neurological outcomes.

Outcome assessment

Neurological outcome at hospital discharge served as the primary endpoint of the study. Neurological outcome at hospital discharge was evaluated using the Cerebral Performance Category (CPC) scale, as originally described by Safar in 1981 [12]. Patients were classified as having a favorable outcome if they achieved CPC scores of 1 or 2, reflecting normal or mildly impaired cerebral function. CPC scores of 3 to 5, which indicate severe neurological disability, persistent coma, or death, were categorized as unfavorable outcomes. Neurological assessments were performed in the absence of ongoing sedation and neuromuscular blockade, and only after the exclusion of major metabolic disturbances.

To ensure the objectivity of outcome determination, clinicians responsible for evaluating patients’ neurological status were blinded to all NSE concentrations and temporal trends. Blinding procedures were implemented to eliminate potential bias arising from knowledge of biomarker levels, thereby preserving the integrity of the outcome assessment process.

Ethical considerations

The study was approved by the Hospital Ethics Committee of County Emergency Clinical Hospital of Sibiu (Approval No. 1971/29.01.2024). Written informed consent was obtained from next of kin or legal representatives, in accordance with institutional policy and the Declaration of Helsinki.

Statistical analysis

Statistical analyses were performed using SPSS (IBM Corp., IBM SPSS Statistics for Windows, Version 25.0. Armonk, NY). Continuous variables were expressed as median with interquartile range (IQR) or mean ± standard deviation, depending on distribution (assessed using Kolmogorov-Smirnov). Comparisons between groups (favorable vs. unfavorable CPC) were performed using the Mann-Whitney U test for non-parametric variables and the chi-square test for categorical variables. Variables included in the multivariable model were selected a priori based on clinical relevance and established prognostic value in post-cardiac arrest outcome studies.

Receiver operating characteristic (ROC) curves were generated for NSE at 24 and 72 hours. Discriminative performance was quantified using the area under the curve (AUC). Optimal prognostic thresholds were identified using Youden’s index. Multivariable logistic regression was performed to evaluate the independent association between NSE levels and neurological outcome, adjusting for clinically relevant covariates. A two-tailed p-value< 0.05 was considered statistically significant.

## Results

Study population

**Figure 1:**
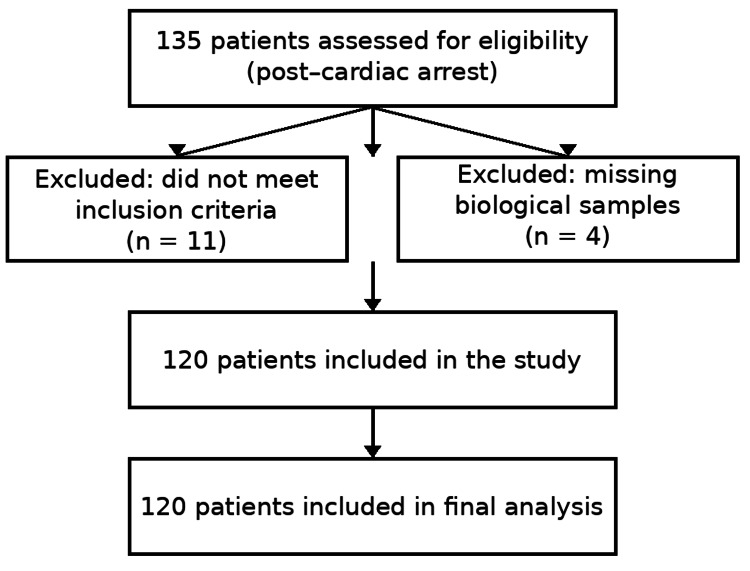
Flowchart of patient selection Flowchart describing patient selection. A total of 135 patients with post–cardiac arrest syndrome were admitted during the study period. Fifteen patients were excluded due to missing samples or failure to meet inclusion criteria, resulting in 120 patients included in the final analysis.

During the 12-month study period, extending from February 2024 to February 2025, a total of 135 patients with post-cardiac arrest syndrome were admitted to the Sibiu County Emergency Clinical Hospital. Among them, 120 individuals fulfilled all prespecified inclusion criteria and were subsequently incorporated into the final analysis (Figure 1). 

The mean age of the study cohort was 72.3 years, reflecting an elderly population with substantial comorbidity burden, and the majority of patients were male, accounting for 68% of the sample. Regarding cardiac arrest characteristics, the initial rhythm was shockable-ventricular fibrillation or pulseless ventricular tachycardia in 41% of cases, while the remainder presented with non-shockable rhythms such as pulseless electrical activity or asystole. Baseline demographic and clinical characteristics are summarised in Table 1.

**Table 1:** Baseline characteristics of the study population. Values are presented as n (%); SD: standard deviation; CPC: cerebral performance category.

Category	Subcategory	Value
Demographics	Age (years)	72.3 ± 10.8 (100%)
	Male	82 (68.3%)
	Female	38 (31.7%)
Arrest location	Out-of-hospital	108 (90.0%)
	In-hospital	12 (10.0%)
Cause of arrest	Cardiac	102 (85.0%)
	Respiratory	12 (10.0%)
	Other	6 (5.0%)
Outcome	Survival to discharge	20 (16.7%)
	CPC 1-2	14 (11.7%)
	CPC 3-4	6 (5%)
	CPC 5	100 (83.3%)

Survival outcomes

Survival outcomes demonstrated the overall severity of illness in this cohort. Only 20 patients, representing 16.7% of the study population, survived to hospital discharge. Neurological outcome assessed at discharge using the Cerebral Performance Category (CPC) scale revealed that 14 patients (11.7%) achieved a favorable neurological status, corresponding to CPC categories 1 or 2. Six patients (5.0%) were discharged with severe neurological disability (CPC 3-4), while the vast majority, 100 patients, or 83.3% died before discharge, corresponding to CPC 5. Overall, 88.3% of the population experienced an unfavorable neurological outcome, underscoring the high mortality and poor functional recovery characteristic of post-cardiac arrest encephalopathy in this setting.

Serum neuron-specific enolase (NSE) levels showed marked differences between outcome groups at both measured time points. At 24 hours after ROSC, patients who ultimately achieved a favorable neurological outcome exhibited a median NSE concentration of 34 ng/mL, with an interquartile range (IQR) of 22-48 ng/mL. In contrast, individuals with unfavorable outcomes had substantially higher NSE values, with a median of 68 ng/mL (IQR 52-94 ng/mL). This difference was statistically significant (p < 0.001), indicating strong early discrimination between groups. By 72 hours post-ROSC, the divergence in biomarker levels became even more pronounced. Favourable-outcome patients demonstrated a median NSE of 28 ng/mL (IQR 20-43 ng/mL), whereas patients with unfavorable outcomes showed a markedly elevated median concentration of 96 ng/mL (IQR 71-128 ng/mL), again with high statistical significance (p< 0.001). These temporal trends suggest a steep and sustained elevation in NSE among patients with progressive hypoxic-ischemic brain injury compared to those with neurological recovery (Tables 2, 3).

**Table 2:** Distribution of NSE categories at 24 and 72 hours by sex. NSE: neuron-specific enolase; values categorized into four predefined ranges, 24 h/72 h: time from return of spontaneous circulation (ROSC), counts represent the number of patients within each NSE category.

NSE Category (µg/L)	Male 24 h (n=82)	Female 24 h (n=38)	Male 72 h (n=82)	Female 72 h (n=38)
<30 µg/L	2 (2.4%)	2 (5.3%)	7 (8.5%)	2(5.3%)
30-60 µg/L	24 (29.3%)	8 (21.1%)	9 (11.0%)	2 (5.3%)
60-90 µg/L	46 (56.1%)	24 (63.2%)	18 (22.0%)	7 (18.4%)
>90 µg/L	10 (12.2%)	4 (10.4%)	48 (58.5%)	27 (71.0%)

**Table 3:** Median NSE levels, interquartile ranges, and p-values by time point and neurological outcome. Median NSE levels and interquartile ranges (IQR) are presented for favorable (CPC 1–2) and unfavorable (CPC 3–5) neurological outcomes at 24 h and 72 h after ROSC. p-values represent comparisons between favorable and unfavorable groups at each time point, demonstrating statistically significant differences in NSE distribution. IQR indicates data variability around the median.

Time point	Outcome group	Median (ng/mL)	IQR (ng/mL)	p-value
24 h	Favorable	34	22-48	<0.001
	Unfavorable	68	52-94	
72 h	Favorable	28	20-43	<0.001
	Unfavorable	96	71-128	

**Figure 2:**
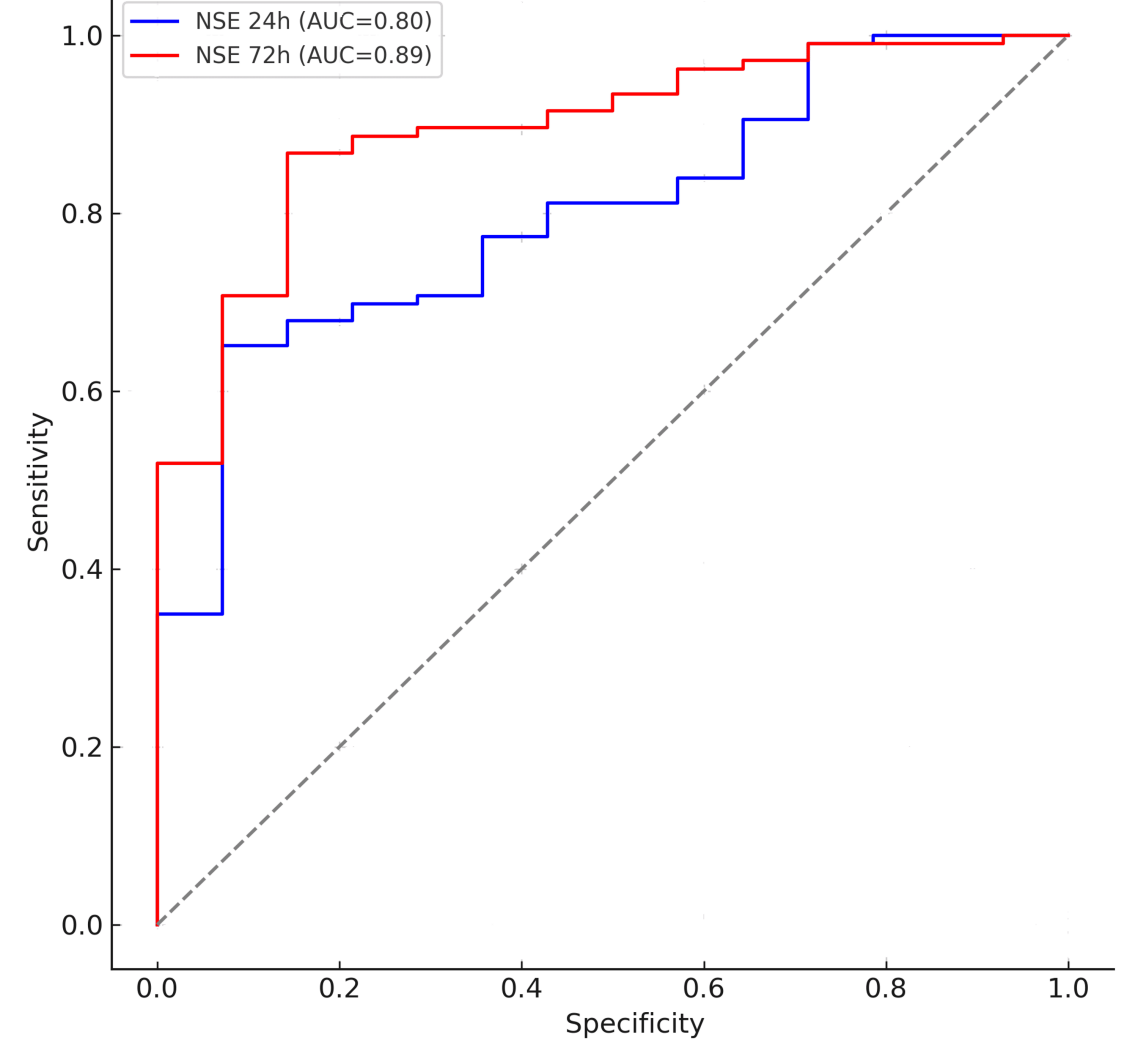
Receiver operating characteristic (ROC) curve for NSE at 24 hours in predicting neurological outcomes. Receiver operating characteristic (ROC) curves for serum neuron-specific enolase (NSE) measured at 24 hours (blue line) and 72 hours (red line) after return of spontaneous circulation (ROSC). The area under the curve (AUC) was 0.80 (95% CI: 0.72-0.89) for NSE at 24 hours and 0.89 (95% CI: 0.83-0.96) for NSE at 72 hours. The 72-hour measurement demonstrated superior discriminative accuracy for predicting unfavorable neurological outcome (CPC 3-5). The diagonal line represents the reference line for a non-informative test. AUC=area under the curve; NSE=neuron-specific enolase; CPC=cerebral performance category; ROSC=return of spontaneous circulation.

**Figure 3:**
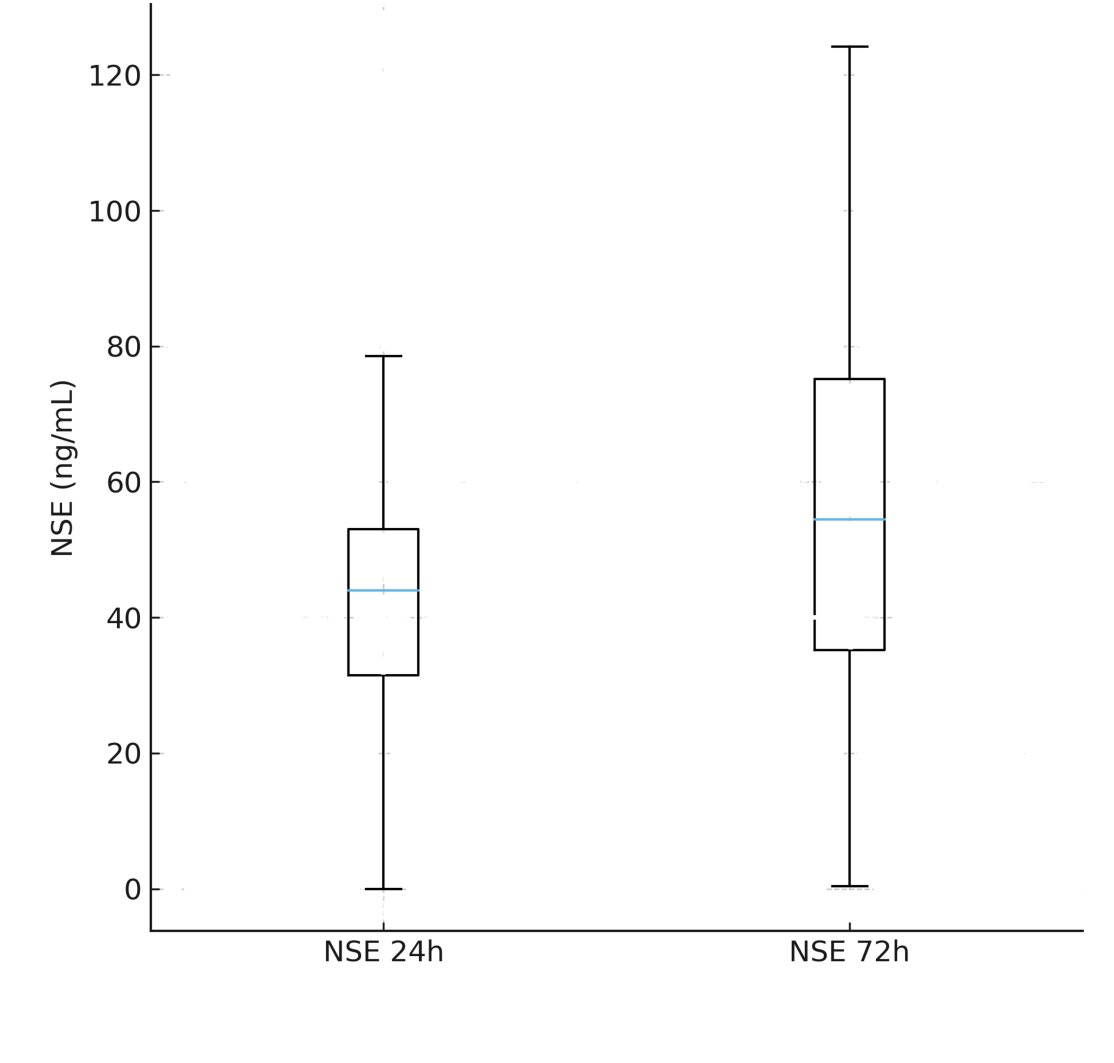
Boxplots showing the distribution of serum neuron-specific enolase (NSE). Boxplots showing the distribution of serum neuron-specific enolase (NSE) concentrations at 24 hours and 72 hours after return of spontaneous circulation (ROSC). NSE values were significantly higher at 72 hours in patients with unfavorable neurological outcome (CPC 3-5), consistent with progressive neuronal injury and improved discriminative performance at the later time point. NSE=neuron-specific enolase.

The predictive performance of NSE for unfavorable outcome was evaluated using receiver operating characteristic (ROC) analysis. NSE measured at 24 hours demonstrated good discriminatory ability, with an area under the curve (AUC) of 0.80. However, the prognostic accuracy improved substantially at 72 hours, where the AUC increased to 0.89, indicating excellent performance for predicting neurological outcome at discharge. Based on Youden’s index, the optimal NSE threshold at 72 hours was identified as 68 ng/mL, which corresponded to a sensitivity of 82% and a specificity of 90% for predicting an unfavorable outcome. This cutoff offers a clinically meaningful balance between false negatives and false positives, reinforcing the utility of late NSE measurement in prognostication. (Figures 2, 3)

Multivariable logistic regression analysis was conducted to adjust for potential confounders, including age and initial cardiac arrest rhythm. After adjustment, higher NSE levels at 72 hours remained independently associated with an unfavorable neurological outcome, with an adjusted odds ratio of 1.12 per 10 ng/mL increase (95% confidence interval 1.05-1.21; p< 0.001). Although shockable rhythm initially appeared to confer protective effects in unadjusted analyses, this variable did not retain statistical significance in the fully adjusted model. NSE at 72 hours thus emerged as the most robust independent predictor of neurological prognosis in this cohort. 

## Discussion

The present prospective study evaluated the prognostic performance of serum neuron-specific enolase (NSE) measured at 24 and 72 hours after return of spontaneous circulation (ROSC) in a cohort of adult post-cardiac arrest patients treated without targeted temperature management (TTM). NSE was measured at 24 and 72 hours after ROSC in accordance with standardized clinical practice and guideline-supported prognostication time points. Although NSE has been reported to peak around 48 hours, measurements at 72 hours are widely accepted to reflect peak or near-peak concentrations and to provide robust prognostic information. Previous work has shown that NSE increases proportionally to the severity of hypoxic-ischemic brain injury, making it one of the most validated biomarkers in this setting [13]. Our findings demonstrate that higher NSE concentrations at both time points were strongly associated with unfavorable neurological outcome (CPC 3-5) at hospital discharge, with NSE at 72 hours showing superior discriminative accuracy (AUC=0.89, 95% CI: 0.83-0.96) compared to measurements at 24 hours (AUC=0.80, 95% CI: 0.72-0.89). These results reinforce the clinical utility of NSE as a biomarker of hypoxic-ischemic brain injury, particularly when measured later in the post-resuscitation period [14]. Our findings are consistent with large registry-based analyses and randomized trials, such as the TTM trial (Nielsen et al. 2013) and the TTM2 trial (Dankiewicz et al. 2021), which have demonstrated the prognostic value of biomarkers, including NSE, across heterogeneous post-cardiac arrest populations [15,16].

Reported optimal cutoff values vary widely (typically 60-90 ng/mL), depending on assay, timing, inclusion criteria, and the use of TTM. The threshold identified in our population, 68 ng/mL at 72 hours, is consistent with values described in clinical practice guidelines and recent validation cohorts, including those examining biomarker trajectories in both TTM-treated and normothermic patients [17].

A distinct feature of our study is that none of the patients received TTM, which likely influenced the absolute NSE levels. Although targeted temperature management was not applied, patients were managed according to standard post-cardiac arrest care with active avoidance of hyperthermia. Core temperature was maintained within the normothermic range in most patients, and no systematic differences in NSE levels according to temperature could be reliably assessed. Hypothermia has been shown to reduce neuronal metabolism, attenuate secondary injury pathways, and delay the release of intracellular proteins such as NSE [18]. Consequently, studies including TTM-treated patients often report lower NSE concentrations at a given time point compared to normothermic cohorts. The absence of TTM in our population may therefore partly explain the relatively high NSE values observed in patients with poor outcomes. Importantly, however, the relative discriminative performance remained robust, suggesting that NSE maintains its prognostic value even in the absence of temperature modulation.

Another relevant aspect is the demographic profile of our cohort. The mean age was relatively high (72.3 ± 10.8 years), and the majority of arrests were out-of-hospital (90%), with a low overall survival rate (17%). 

The clinical implications of our findings are noteworthy. NSE, particularly at 72 hours, may serve as a valuable component of multimodal prognostication in settings where TTM is not routinely implemented. However, NSE should never be used in isolation to guide decisions regarding withdrawal of life-sustaining therapy. Instead, it should complement neurological examination, electroencephalography, neuroimaging, and other prognostic tools. The identification of a population-specific cutoff may support early counseling of families and optimize post-resuscitation care pathways. 

This study has several strengths, including its prospective design, standardized timing of biomarker sampling at predefined intervals, and the inclusion of all eligible patients who survived long enough to complete biomarker assessment, including those who died shortly after the 72-hour time point, thereby minimizing survivorship bias. Nonetheless, several limitations must be acknowledged. The study was conducted in a single center, which may limit generalizability. The sample size, although comparable to similar studies, remains modest. We did not analyze additional biomarkers such as S100B, neurofilament light chain (NfL), or tau proteins, which could provide complementary information. The absence of TTM, although relevant to real-world practice in many centers, may limit comparability with literature from high-resource settings, where TTM remains the standard. The incomplete documentation of certain clinical parameters inherent to the post-cardiac arrest setting limited their inclusion in baseline analyses.

In summary, our study demonstrates that NSE measured at 72 hours is a reliable predictor of neurological outcome in post-cardiac arrest patients managed without TTM. The biomarker exhibited strong discriminative performance and identified patients at high risk of unfavorable outcomes with good sensitivity and specificity. Integration of NSE into a comprehensive multimodal prognostic algorithm may help refine clinical decision-making in the post-resuscitation period.

## Conclusions

In this prospective study of post-cardiac arrest patients managed without targeted temperature management, serum neuron-specific enolase (NSE) demonstrated good prognostic performance for hypoxic-ischemic brain injury. NSE concentrations at both 24 and 72 hours were higher in patients with unfavorable neurological outcomes, with the 72-hour measurement showing superior discriminative accuracy (AUC=0.89) and an optimal cutoff of 68 ng/mL for identifying high-risk patients.

These findings support the use of NSE as part of a multimodal prognostic strategy alongside clinical examination, neurophysiological testing, and neuroimaging. Further multicenter studies with larger cohorts are warranted to validate these results and refine prognostic thresholds in normothermic post-cardiac arrest care.
